# Fourier domain diffuse correlation spectroscopy with heterodyne holographic detection

**DOI:** 10.1364/BOE.400525

**Published:** 2020-10-28

**Authors:** Edward James, Samuel Powell

**Affiliations:** 1 Department of Medical Physics & Biomedical Engineering, University College London, London, WC1E 6BT, UK; 2 Faculty of Engineering, The University of Nottingham, University Park, Nottingham, NG7 2RD, UK

## Abstract

We present a new approach to diffuse correlation spectroscopy which overcomes the limited light throughput of single-mode photon counting techniques. Our system employs heterodyne holographic detection to allow parallel measurement of the power spectrum of a fluctuating electric field across thousands of modes, at the shot noise limit, using a conventional sCMOS camera. This yields an order of magnitude reduction in detector cost compared to conventional techniques, whilst also providing robustness to the effects of ambient light and an improved signal-to-noise ratio during *in vitro* experiments. We demonstrate a GPU-accelerated holographic demodulation system capable of processing the incoming data (79.4 M pixels per second) in real-time, and a novel Fourier domain model of diffuse correlation spectroscopy which permits the direct recovery of flow parameters from the measured data. Our detection and modelling strategy are rigorously validated by modulating the Brownian component of an optical tissue phantom, demonstrating absolute measurements of the Brownian diffusion coefficient in excellent agreement with conventional methods. We further demonstrate the feasibility of our system through *in vivo* measurement of pulsatile flow rates measured in the human forearm.

## 1. Introduction

Diffuse correlation spectroscopy (DCS) is an optical imaging modality in which the ensemble average of flow within a tissue sample can be inferred from measurement of the intensity autocorrelation function of light that has passed through the sample. Under optical properties typical of biological tissues, measurements are sensitive to flow at a depth approximately equal to one half to one third of the optical source-detector separation (SDS) distance [1,2]. Thus for a typical DCS setup with an SDS distance of 2-3 cm, attempts to measure cerebral blood flow (CBF) are limited to superficial cortical regions. To date, deep CBF has not been measured using DCS in human studies [1].

Conventional implementations of DCS typically employ single mode photon counting techniques. Such methods are limited by low light throughput [1] in a single mode, placing a minimum limit on the detection time [3]. Increasing penetration depth requires the use of larger SDS distances, which will decrease the available signal-to-noise ratio (SNR) further (by increasing the number of absorption and scattering events, since the attenuation of near-infrared (NIR) light by these two mechanisms is in the order of 10 dB/cm [4]).

Increasing acquisition time can ameliorate this situation but leads to a reduction in temporal resolution. Taking the average of many single-mode detection fibres bundled together is an expensive option that requires many photon counting detectors and can increase complexity of system integration. Improved collection optics, the use of few-mode detection fibres, and increasing the amount of light delivered to the tissue can also help to improve SNR [1,5,6]. However, patient safety limits must be adhered to, which necessitates an optical source of sufficiently large diameter and low power rating. All of the above characteristics limit the applicability of conventional homodyne DCS in portable continuous monitoring applications to which optical methods are otherwise well suited.

### 1.1 Motivation

Previous authors have noted that improvements to increase SNR and temporal resolution will help the development of DCS functional experiments, as well as expand the range of uses for DCS in clinical monitoring [1]. The utility of high frame rate (∼20 Hz) DCS measurements, compared to the frame rates of 0.3 to 1 Hz that have historically been used, has also been discussed [7]. These benefits include improved monitoring of cerebrovascular autoregulation dynamics, more robust identification of motion artefacts, and increased measurement throughput that could enable high spatial resolution with fewer detectors. Improvements in SNR are also motivated by the ability to perform flow measurements with improved spatial resolution and depth penetration through the use of acousto-optic tomography (AOT) [3,8–10], which is an emerging hybrid imaging modality that makes use of the ultrasound modulation of light [11]. The modulation efficiency of photons by ultrasound is low at biologically safe power levels, and therefore significantly improved SNR is required in order to detect this modulated component [9].

An interferometric diffusing wave spectroscopy (iDWS) system has recently been described which makes use of multimode fibre detection and a high speed line-scan CMOS camera capable of operating at 333 kHz [12]. Although this technique is robust to the effects of ambient light, its sample rate is considerably slower than a conventional diffusing wave spectroscopy (DWS) system (which is typically in the order of ∼10 MHz) and it cannot resolve sample decorrelations shorter than 6 μs. Additionally, this approach does not have the temporal resolution necessary to sufficiently resolve ultrasound tagged photons, which are typically modulated by ultrasound pressure fields fluctuating in the range of 1 - 5 MHz [3].

The use of a multi-pixel interferometric diffuse correlation spectroscopy (iDCS) system has also been presented, which uses a design wavelength of 1064 nm in order to improve SNR and depth sensitivity. Compared to a more commonly used wavelength of 785 nm, this system encounters less optical scattering events and also benefits from an increase in photon availability [13]. Maximum permissible exposure limits are also higher at 1064 nm, meaning that roughly four times more optical power can be used. However, this technique is marred by bulky equipment (cryostats associated with superconducting nanowire photon detectors) or afterpulsing (InGaAs single photon avalanche diodes), which prohibit the clinical application or accuracy of this technique at present, respectively [14].

### 1.2 Holographic techniques

In this work, we explore an alternative approach to DCS measurements that employs a holographic method known as heterodyne parallel speckle detection (HPSD). HPSD has been well described in the literature since its inception in 2003 [15–18]. The experimental configuration of an HPSD system consists of a Mach-Zender interferometer, where the reference and sample arms are recombined and interfere on a digital camera. Temporal filtering occurs over the camera integration period, and the resulting images record the first-order power spectral density of the scattered electric field, $S_{1}$, at a particular frequency and with a certain bandwidth. By detuning the frequency of the reference arm of the interferometer by a pair of Bragg cells, $S_{1}$ may be sampled at frequency shifts (Δf) from that of the input optical field, allowing the frequency spectrum of the scattered light to be acquired. The heterodyne gain and shot noise limited performance [17] of this technique permit illumination below the maximum permissible exposure limits of tissue, and it is therefore particularly suited to *in vivo* flow detection [8]. HPSD has previously been used to measure convective flow rates *in vitro*, by fitting measured power spectra to a Fourier domain DWS model of convective motion [19]. This group quantified diffusive Brownian motion by fitting measured data to the discrete Fourier transform of a DWS model of Brownian motion. The same study also measured localised superficial microvascular convective flow rates in the cerebral cortices of 2 mice and the retinas of 3 adult rats by imaging through a 5 mm × 5 mm aperture. However, this study used a camera exposure time of 30 ms and a camera frame rate of 12 Hz, which provided insufficient temporal resolution to resolve pulsatile flow *in vivo*, especially after frame averaging. Additionally, it is well documented in the DCS literature that a Brownian diffusion model, rather than a convective motion model, provides a better fit to measured DCS data over a broad range of tissue types [2,20].

Using HPSD to measure flow, at rates sufficient to resolve pulsatile information, brings significant challenges. To employ holographic DCS in a practical *in vivo* setting requires that we use short exposure times in order to minimise the effects of sample movement and external sources of vibration which disrupt the interferometric configuration. Additionally, short exposure times are required to facilitate a high parameter output rate that can resolve fast pulsatile changes. However, reducing the exposure time comes at the cost of a wider instrument response function (IRF), which will result in a broadening of the measured power spectra, which in turn will increase the complexity of data sampling and interpretation. A related complication arises as whilst the true field autocorrelation, $G_{1}$, and power spectral density, $S_{1}$, of the scattered light form a Fourier transform pair, the data measured using conventional and holographic DCS systems will deviate due to differences in the nature of the measurement systems. These differences arise due to the nature of the sampling in the two domains, the effects of IRF broadening and static scatterers, and the differing effects of measurement noise in the two configurations. Whilst our technique offers many advantages compared to conventional measurement techniques, data acquisition speed and processing load requirements are high, especially if we wish to achieve real-time data acquisition at high parameter output rates. There is also a trade-off to consider between speed of data acquisition, making use of averaging to improve SNR, and sampling the measured power spectra at a sufficient number of frequency shifts so as to permit robust fitting to a forward model.

### 1.3 Overview and contribution

We present a high speed Fourier domain HPSD implementation of DCS suitable for making real-time *in vivo* measurements at a frame rate that permits the recovery of pulsatile flow. Our approach employs HPSD to allow averaging over thousands of modes to realise improvements in SNR (which is demonstrated *in vitro* in this paper), and operates with continuous wave (CW) illumination without disturbance from ambient light.

In Section 2. we develop a novel Fourier domain DCS model which allows for parameter fitting in the native domain of the data, permitting extraction of flow parameters whilst respecting the nature of the noise in the measurement system. Furthermore, consideration of the IRF of the holographic DCS instrument allows us to appropriately optimise our detection strategy to allow for high parameter output rates. In Section 3. we describe the architecture of our holographic DCS instrument which uses a highly parallel GPU-accelerated holographic demodulation pathway to manage the processing requirements for the technique; we demonstrate measurements of the power spectral density of the electric field across ∼1,300 speckles at 6 discrete frequency shifts with an overall parameter output rate of 23.8 Hz. In Section 4. we show that our novel Fourier domain DCS model provides accurate absolute interpretation of measured data in their native domain, both at room temperature and over a physiologically relevant temperature range. We also show that our instrument offers robustness to the effects of ambient light, and describe how the improvement in the SNR of our $S_{1}$ measurement scales with the square root of the number of detected camera pixels *in vitro*. Finally, we demonstrate the *in vivo* application of our inexpensive camera based detection system by recovering pulsatile flow rates measured in the human forearm.

## 2. Theory

DCS is used to infer the mean-square particle displacement, $\left\langle \Delta r^{2}(\tau )\right\rangle$, of tissue scatterers in the illuminated region between an optical source and detector through measurement of the temporal fluctuation of NIR light that has passed through a sample [20,21]. For the case of deterministic convective motion of scatterers, $\langle \Delta r^{2}(\tau )\rangle =\langle V^{2}\rangle \tau^{2}$, where $\left\langle V^{2}\right\rangle$ (${\mathrm{cm}}^{2}$/$s^{2}$) is the second moment of the speed distribution of scattering particles. In the case of diffusive Brownian motion of scatterers $\langle \Delta r^{2}(\tau )\rangle =6D_{b}\tau$, where $D_{b}$ (${\mathrm{cm}}^{2}$/s) is the effective Brownian diffusion coefficient of scattering particles [22]. $D_{b}$ can be used as a blood flow index (BFI) parameter in biological tissues, although it has units of ${\mathrm{cm}}^{2}$/s, rather than the more commonly encountered blood perfusion unit of ml/min/100g [23]. Relative change in blood flow ($rBF=BFI/BFI_{0}$, where BFI_0_ is the baseline measurement of BFI) measured by DCS has been shown to agree with relative changes in absolute blood flow as measured by gold standard techniques, such as arterial spin labelling magnetic resonance imaging (ASL-MRI) [1]. To determine the pertinent parameters from the measured data requires fitting to a model which considers the geometry under consideration. In this section we will review the conventional approach to this problem (which is performed in the time domain), before developing a Fourier domain approach suitable for use with the HPSD technique.

### 2.1 Conventional DCS

**Fig. 1.**
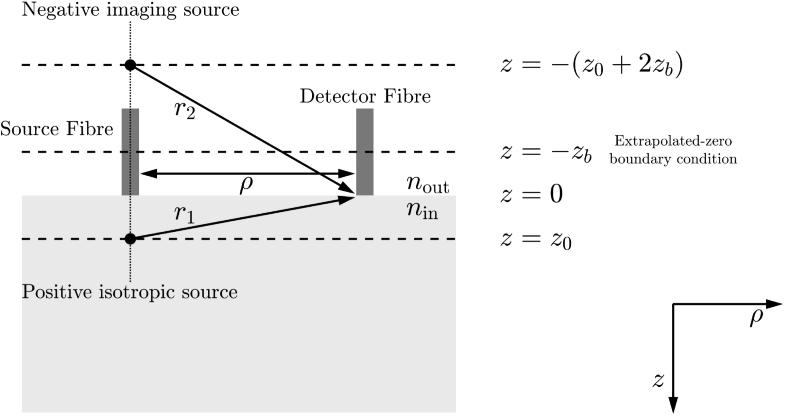
Semi-infinite geometry reflection mode model and notation used in DCS measurements. Adapted from [20].

Boas and Yodh [24] employed correlation transport theory to derive the correlation diffusion equation, which describes the propagation of the temporal electric field autocorrelation function in turbid biological tissue. In a clinically relevant semi-infinite geometry (Fig. 1) [7], the *un-normalised* solution for this autocorrelation function for dynamic scatters is given by

(1) $$G_{1d}(\tau )=\frac{S_{0}}{4\pi D}\left[\frac{\exp^{-K(\tau )r_{1}}}{r_{1}}-\frac{\exp^{-K(\tau )r_{2}}}{r_{2}}\right],$$

where:

• τ is the delay (or lag) time of the autocorrelation function;
• $S_{0}$ is the optical source intensity;
• D is the optical diffusion coefficient, $\frac{1}{3(\mu_{a}+\mu_{s}^{\prime })}$, $\mu_{a}$ is the absorption coefficient and $\mu_{s}^{\prime }$ is the reduced scattering coefficient;
• $K(\tau )=\sqrt{3\mu_{a}\mu_{s}^{\prime }+\mu_{s}^{\prime 2}k_{0}^{2}\langle \Delta r^{2}(\tau )\rangle }$ is the decay constant, $k_{0}$ is the wavevector magnitude of the incident CW light field, 2πn/λ, and λ is the wavelength of the CW light field;
• $z_{0}=1/\mu_{s}^{\prime }$ is the depth into the medium at which the collimated source is approximated as a positive isotropic source;
• ρ is the distance between the optical source and detector;
• $R_{eff}=-1.440n^{-2}+0.710n^{-1}+0.668+0.0636n$ is the effective reflection coefficient and accounts for the reflective index mismatch between air ($n_{out}$) and tissue ($n_{in}$), where $n=n_{in}$/$n_{out}$. This is a commonly used series approximation of [2]
  (2) $$R_{eff}=\frac{R_{\phi }+R_{j}}{2-R_{\phi }+R_{j}},$$
  where $R_{\phi }$ and $R_{j}$ are the isotropic fluence rate and directional flux terms, respectively [25];
• $z_{b}=\frac{2z_{0}}{3}\frac{\left(1+R_{eff}\right)}{\left(1-R_{eff}\right)}$, $-z_{b}$ is the position at which there should be a signal size of zero in order to fulfil the extrapolated boundary condition [25];
• $r_{1}=\sqrt{z_{0}^{2}+\rho^{2}}$ is the distance between the detector and an approximated positive isotropic imaging source;
• $r_{2}=\sqrt{{\left(2z_{b}+z_{0}\right)}^{2}+\rho^{2}}$ is the distance between the detector and an approximated negative isotropic imaging source located at position $z=-(z_{0}+2z_{b})$.

The normalised temporal electric field autocorrelation function is then [23]

(3) $$g_{1d}(\tau )=\frac{G_{1d}(\tau )}{G_{1d}(\tau =0)}.$$

In practice the normalised intensity temporal autocorrelation function ($g_{2}$) at the detector is directly measured, where

(4) $$g_{2}(\tau )\equiv \frac{\left\langle I(t)I(t+\tau )\right\rangle }{\langle I(t)\rangle^{2}},$$

and where intensity is proportional to the time average of the square of the electric field, $I(t)=|E(t){|}^{2}$. Assuming a zero mean Gaussian electric field, the Siegert relation can then be used to extract $g_{1}(\tau )$ from $g_{2}(\tau )$ [26]

(5) $$g_{2}(\tau )=1+\beta |g_{1}(\tau ){|}^{2},$$

where [24]

(6) $$g_{1}(\tau )=\alpha |g_{1d}(\tau )|+(1-\alpha ),$$

and where α∈[0,1] and β∈[0,1] are both unitless factors.

The parameter α is an adaptation to biological tissue and refers to the fraction of scattering events due to dynamic, rather than stationary, scatterers. This factor is therefore the ratio of moving scatterers to the total number of scatterers in a sample. The parameter β is inversely proportional to the number of detected speckles or modes, and is also related to the coherence length and stability of the laser light source, as well as the number of detected polarisation states [22,24]; it can be determined from the *y*-intercept of $g_{2}(\tau )$ at τ=0.

### 2.2 Fourier domain DCS

It is well established according to Weiner-Khinchin theorem that the first-order Doppler spectrum, $s_{1d}(\omega )$, and $g_{1d}(\tau )$ are a Fourier transform pair [8,19,24,27–29]

(7) $$s_{1d}(\omega )=\int_{-\infty }^{+\infty }g_{1d}(\tau )\exp^{-i\omega \tau }\;d\tau .$$

The Fourier transform of Eq. (1) is then

(8) $$S_{1d}(\Delta \omega )=\frac{S_{0}}{4\pi D}\left[F(\Delta \omega ,r_{1})-F(\Delta \omega ,r_{2})\right],$$

where Δω is angular detuning frequency. In the case of Brownian motion and Δω>0, we find that

(9) $$\begin{array}{rl} F_{\left[D_{b}\right]}(\Delta \omega_{+},r)=\frac{1}{\Delta \omega^{3/2}} & \left(\frac{1}{4}+\frac{i}{4}\right)\mu_{s}^{\prime }\sqrt{6C}\exp\left(-\frac{D(\Delta \omega )}{2}-\frac{E(\Delta \omega )}{2}\right)\times \ldots \\ & \left(\exp\left(D(\Delta \omega )\right)\text{erfc}\left(A_{-}(\Delta \omega )\right)-i\exp\left(E(\Delta \omega )\right)\text{erfc}\left(A_{+}(\Delta \omega )\right)\right), \end{array}$$

where the auxiliary function

(10) $$A_{\pm }(\Delta \omega )=\frac{(2\mp 2i)\Delta \omega \sqrt{\frac{\mu_{a}\mu_{s}^{\prime }}{2C}}+(1\pm i)\mu_{s}^{\prime 2}r\sqrt{6C}}{2\sqrt{2}\mu_{s}^{\prime }\sqrt{\Delta \omega }},$$

erfc is the complementary error function,

(11) $$C=k_{0}^{2}D_{b},$$

(12) $$D(\Delta \omega )=\frac{i\mu_{a}\Delta \omega }{\mu_{s}^{\prime }C},$$

and

(13) $$E(\Delta \omega )=\frac{3i\mu_{s}^{\prime 2}Cr^{2}}{\Delta \omega }.$$

We note that $g_{1d}(\tau )$ is an even and real function, and therefore its Fourier transform is also real. For convective motion we find that

(14) $$F_{\left[V^{2}\right]}(\Delta \omega ,r)=\sqrt{\frac{3\mu_{a}\mu_{s}^{\prime }}{\mu_{s}^{\prime 2}k_{0}^{2}V^{2}r^{2}+\Delta \omega^{2}}}K_{1}\left(\sqrt{3\mu_{a}\left(\mu_{s}^{\prime }r^{2}+\frac{\Delta \omega^{2}}{\mu_{s}^{\prime }k_{0}^{2}V^{2}}\right)}\right),$$

where $K_{1}$ is the first modified Bessel function of the second kind.

## 3. Methods

### 3.1 Heterodyne parallel speckle detection

In general, interferometry techniques involve the recombination of a reference beam with a signal beam that has been transmitted through a sample. The source is split into two parts by a beamsplitter to form a signal beam and a reference beam. In our implementation of an HPSD interferometry system, the frequency of the signal beam is unshifted, such that $\omega_{S}=\omega_{L}$, where $\omega_{S}$ is the optical frequency of the signal beam, and $\omega_{L}$ is the optical frequency of the laser source beam. The reference beam, $\omega_{R}$, is shifted away from $\omega_{L}$ using a pair of polarisation-independent acousto-optic modulators (AOMs) with a centre wavelength of 150 MHz. A pair of AOMs is used as the required frequency shift can be very small compared to the centre wavelength of the AOMs [3], and thus one AOM is used to produce a negative frequency shift ($\omega_{AOM1}$), and the other AOM a positive frequency shift ($\omega_{AOM2}$) of a slightly different magnitude. This results in $\omega_{R}=\omega_{L}+\omega_{AOM1}+\omega_{AOM2}$.

Having passed through the sample, the signal field takes the form $E_{S}(t)=E_{S}\exp^{i\omega_{S}t}$. The reference field takes the form of $E_{R}(t)=E_{R}\exp^{i\omega_{R}t}$. The sample and reference beams are recombined in a beamsplitter and are interfered on a camera sensor. This recombination occurs slightly off-axis with respect to the camera sensor by a small tilt angle [16].

The intensity of the speckle interference pattern that is detected by the camera is then

(15) $$I(t)=|E_{S}(t)+E_{R}(t){|}^{2},$$

which expands to [16]

(16) $$I(t)=|E_{S}{|}^{2}+|E_{R}{|}^{2}+E_{S}E_{R}^{*}\exp^{-i(\omega_{R}-\omega_{S})t}+\;E_{S}^{*}E_{R}\exp^{+i(\omega_{R}-\omega_{S})t},$$

where the first two terms of Eq. (16) correspond to the self-beating homodyne terms, and the third and fourth terms correspond to the heterodyne signal-reference cross terms. Therefore the strength of the measured signal (i.e., the two heterodyne terms) depends on both the transmitted signal beam and the reference beam, according to

(17) $$G=\frac{|E_{S}E_{R}|}{|E_{S}{|}^{2}}\gg 1,$$

where G is heterodyne gain [30]. The use of a large reference beam intensity allows this technique to reach the shot noise limit, permitting optimum acquisition times [3,9], enabling its use for *in vivo* imaging [8].

For a given value of $\omega_{R}$, $N_{f}$ camera frames are captured using a given camera exposure time, $\tau_{e}$, and camera frame rate, $f_{s}$. The off-axis recombination allows the spatial separation of the zero order of diffraction and the two heterodyne gain terms (which are a conjugate pair). This facilitates the *spatial* filtering component of HPSD, owing to the separation of the signal, shot noise, speckle decorrelation noise and technical noise of the reference beam in the spatial frequency domain of the detected interference pattern (Fig. 3) [8]. As well as this spatial filtering, *temporal* filtering is also achieved by two methods. An inherent temporal filter is applied due to the integration time of the camera. Further temporal filtering is also achieved by constructing a hologram in the camera plane from two or more consecutive images. For example, using a DC subtraction temporal filtering method, the camera plane hologram, $H_{C}$, is constructed as the difference of two successive images (i.e., $N_{f}=2$)

(18) $$H_{C}=I_{1}-I_{2},$$

which removes the two homodyne terms, $|E_{S}{|}^{2}$ and $|E_{R}{|}^{2}$ in Eq. (16), from the hologram. The intensity hologram, $H_{R}$, is then reconstructed in the object plane by performing a 2D discrete Fourier transform of the camera plane hologram [31]

(19) $$H_{R}=|F_{2D}(H_{C}){|}^{2}.$$

A masking operation can then be performed to sum over the two heterodyne signal terms and also to sum over a shot noise mask, which is implemented in one of the two ‘quiet’ corners of the reconstructed hologram (Fig. 3). The *average* pixel value in each mask is then obtained, which we denote by $S_{\pm \Delta \omega }$ for the two heterodyne masks, and N for the shot noise mask. In order to avoid contamination by the technical noise of the reference beam, none of the three masks should be placed in the low spatial frequency region of $H_{R}$ [17]. The unnormalised first-order power spectral density at a given detuning frequency may then be calculated for each heterodyne term as [19,32]

(20) $$S_{1_{\left(\pm \Delta \omega \right)}}=\frac{S_{\pm \Delta \omega }}{N}-1.$$

Phase-shifting holography (which is distinct from the DC subtraction temporal filtering method) involves offsetting one of the AOM detuning frequencies by $f_{s}/N_{f}$, so that multiple images of the interference pattern can be recorded which have slightly different phase offsets between the signal and reference beams [3]. This has the effect that, following temporal filtering, peak sensitivity to the first heterodyne gain term of the IRF will be at DC (Fig. 5). For phase-shifting with $N_{f}=2$, $H_{C}$ is constructed according to Eq. (18), whilst for phase-shifting with $N_{f}=4$, $H_{C}$ is constructed as [16]

(21) $$H_{C}=(I_{1}-I_{3})+i(I_{2}-I_{4}).$$

Four-frame phase-shifting holography allows separation of the two heterodyne gain terms in the reconstructed hologram; however, this comes at the cost of increased acquisition time, which one group argues should ideally be less than the speckle decorrelation time of the sample [8]. However, another group argues that this ‘decorrelation problem’ does not exist, and that optimal sensitivity can be achieved by using a camera exposure time in the order of the speckle decorrelation time of the sample [30]. Compared to other more complicated multiple-phase-shifting techniques, it has been shown that a DC subtraction technique is sufficient to reach the shot noise limit [33]. This shot noise limited performance was verified for our HPSD system (Fig. 3). The interested reader is referred to [16] for further information regarding the formation of multiple frame holograms.

The expression for the IRF for the first and second heterodyne terms of an HPSD instrument is given by [15,28]

(22) $$B_{\pm \Delta \omega }={\left|sinc\left(\frac{\Delta \omega }{2\pi }\tau_{e}\right)\sum_{k=1}^{k=N_{f}}\exp(-2ik\pi /N_{f})\exp(\mp 2ik\pi \Delta \omega /\omega_{s})\right|}^{2},$$

where $\omega_{s}$ is the angular frame rate of the camera and

(23) $$sinc(t)=\frac{sin(\pi t)}{\pi t}$$

is the normalised sinc function.

The total normalised measured response in the Fourier domain is then [28]

(24) $$s_{1}(\Delta \omega )=\alpha s_{1d}(\Delta \omega )*B_{\pm \Delta \omega }+(1-\alpha )B_{\pm \Delta \omega },$$

where ∗ is the convolution product. If the IRF is much narrower than the Doppler broadening that is being measured, Eq. (24) can be simplified to [15,19]

(25) $$s_{1}(\Delta \omega )=\alpha s_{1d}(\Delta \omega )+(1-\alpha )B_{\pm \Delta \omega }.$$

If we make the assumption that a sample is composed entirely of dynamic scatterers, then Eq. (25) becomes

(26) $$s_{1}(\Delta \omega )=s_{1d}(\Delta \omega ).$$

Likewise, if a sample is composed entirely of static scatterers, then Eq. (25) becomes

(27) $$s_{1}(\Delta \omega )=B_{\pm \Delta \omega }.$$

The naïve approach to recovering flow information from Fourier domain DCS data is simply to transform the data into the time domain, and use established theory to fit for $D_{b}$. However, although the data are fundamentally equivalent, this approach will lead to errors owing to differences in the sampling of the data in the time and frequency domains, the effects of static scattering in a Fourier domain DCS system, the nature of the measurement noise, and broadening by the IRF.

### 3.2 System design and integration

We have developed a new Fourier domain implementation of DCS by building an off-axis HPSD instrument. In our instrument, a pair of AOMs is placed on the reference arm (Gooch & Housego, Fiber-Q, 150 MHz centre frequency, 785 nm, upshift & downshift). The signal field that has been scattered by the sample is collected in a reflection mode geometry through the aperture of a ∅ 5.0 mm core liquid light guide (Thorlabs, LLG5-4Z), which has a numerical aperture of 0.52 (we note that using a multimode detector fibre is an alternative approach which could be implemented with modified detection optics). Our sCMOS camera (FLIR, BFS-U3-16S2M-CS) has a sensor size of 1440 × 1080 pixels, which we truncate to $2^{n}$ × $2^{n}$ (where n is an integer between 3 and 10) so as to facilitate holographic reconstruction by the fast Fourier transform algorithm, and we denote $N_{pix}=2^{n}$.

In order to maximise the use of the square camera sensor layout whilst employing a circular aperture, the maximum achievable diameter of one heterodyne term on the camera sensor can be shown to be $N_{pix}\sqrt{2}/(3+\sqrt{2})$ pixels, so as not to collect any signal at the zero order DC term, whilst ensuring that the aperture is fully contained within the reconstructed hologram (i.e., without spatial aliasing). This is equivalent to calculating the maximum diameter of four equally sized circles that are both aligned along the diagonal of a square and fully contained within the square.

**Fig. 2.**
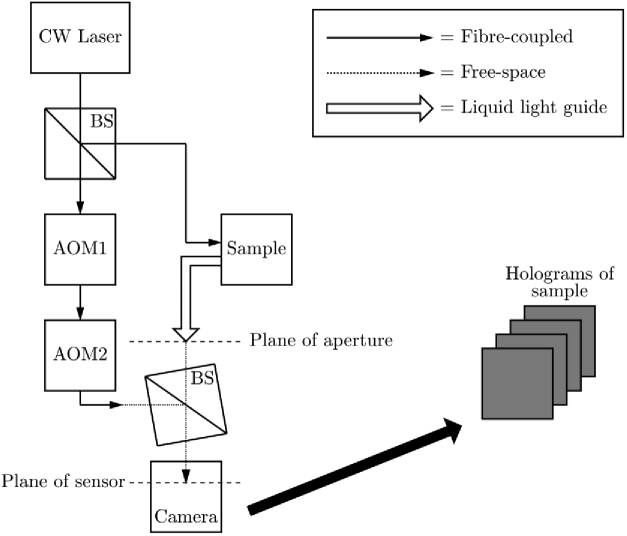
Schematic representation of our off-axis HPSD system. A continuous wave (CW) laser source is split into a reference arm and a sample arm in a fibre-coupled beamsplitter (BS). The reference arm is frequency shifted by a pair of acousto-optic modulators (AOM1 and AOM2). Light is collected from the sample in a reflectance mode geometry through the aperture of a liquid light guide. The two arms are recombined off-axis in a cube BS.

We wish to ensure that each speckle illuminates no less than one camera pixel, in order to prevent multiple out of phase speckles illuminating a single pixel. Our camera has a pixel size, $\Delta_{pix}$, of 3.45 μm. Therefore we aim to constrain the minimum speckle size, S, according to [34,35]

(28) $$S=\frac{{\left(\lambda z\right)}^{2}}{A_{\text{aperture}}}\geq \Delta_{pix}^{2},$$

where z is the observation distance between the plane of the aperture and the plane of the camera sensor (as depicted in Fig. 2), and $A_{\text{aperture}}$ is the area of the aperture. This yields a minimum observation distance, $z_{min}$, of 19.5 mm. Additionally, by considering the maximum spatial frequency that can be resolved at the detector, it has also been shown that [36]

(29) $$z_{min}=\frac{\sqrt{2}\Delta_{pix}L}{\lambda },$$

where L is the characteristic dimension of the aperture. This expression yields a value of $z_{min}$ = 31.1 mm for our system. The experiments reported in this paper use a value of z = 75 - 80 mm, which adheres to both of these constraints, and for which a single speckle occupies the area of ∼15 - 17 square pixels, respectively. We sample ∼5,200 speckles in each signal mask for $N_{pix}=1,024$, and ∼1,300 speckles for $N_{pix}=512$. Decreasing the value of z from 75 - 80 mm to 31.1 mm would increase the number of detected speckles on the camera sensor, but this would lead to sampling of the zero order DC term in the centre of the reconstructed hologram and is therefore avoided.

**Fig. 3.**
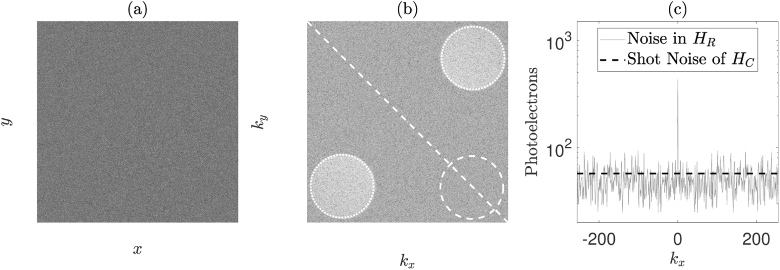
(a) Camera plane hologram, $H_{C}$, formed using DC subtraction temporal filtering. (b) Arbitrary logarithmic representation of a reconstructed intensity hologram, $H_{R}$. The two heterodyne gain terms, $S_{\pm \Delta \omega }$, are masked by the dotted circles (which are a conjugate pair), the shot noise mask, N, is depicted by the dashed circle. (c) The thin grey solid line shows the value of the diagonal white dashed line that has been superimposed on $H_{R}$, averaged over ±5 pixels in $k_{y}$. The thick dashed black line shows the average shot noise value of all the pixels in $H_{C}$ for this particular image reconstruction.

**Fig. 4.**
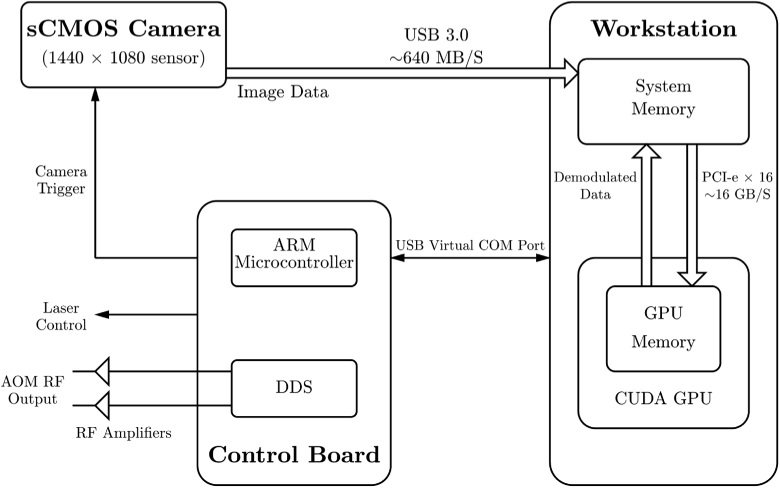
Integrated system architecture and data streaming via a highly parallel GPU-accelerated demodulation pathway. The control board synchronises experiments, holograms are relayed from the camera to the workstation’s system memory, and custom CUDA kernels implement the holographic demodulation process.

Real-time operation of our Fourier domain instrument is enabled by a GPU-accelerated holographic demodulation system, which currently operates with a throughput of 79.4 M pixels per second for $N_{pix}=512$. One of the main challenges for the application of holographic DCS at high frame rates is fast data acquisition and processing load: calculating an individual $S_{1}$ measurement requires frequency shifting, image capture, temporal filtering, 2D fast Fourier transform, spatial filtering and reduction. We have therefore designed a custom integrated holographic DCS system with a high throughput, with the aim of achieving real-time demodulation and data acquisition at fast imaging frame rates, a schematic of which is shown in Fig. 4. The system consists of a camera, a workstation and a control board. The control board receives instructions from the workstation via a USB virtual COM port, and carries out the following three functions:

(1) generation of radio frequency (RF) waveforms for the AOMs using direct digital synthesis (DDS);
(2) control of the laser output;
(3) synchronisation of the overall experiment by generation of camera triggers whilst performing sweeps through the required frequency shifts.

Image data is then relayed from the camera to the workstation via a high bandwidth USB 3.0 connection, and then to the GPU via a 16 lane PCI express interface. Custom CUDA kernels implement the holographic demodulation pathway (i.e., calculation of $S_{1}$ values from raw camera frames, as described in Section 3.1) using the CUDA FFT library. Demodulated data are passed back to the workstation’s system memory for analysis. The use of this highly parallel GPU-accelerated demodulation pathway, together with our tightly synchronised instrument with minimal dead-time between frequency shifts, allows us to rapidly measure the power spectral density of light scattered by a diffusing sample at a high parameter output rate (i.e., ∼20 Hz [7]) in real-time.

## 4. Experiments and results

All experiments were performed using a 785 nm CW long coherence length (∼9.5 m) laser with a linewidth of ∼10 MHz (iBeam Smart S WS, Toptica), with a maximum optical output power of 120 mW. Conventional DCS experiments used single-mode fibre detection to couple the sample to a single photon avalanche detector, which has a typical dead time of 45 ns and a response time of 30 ns (COUNT-50N, Laser Components), giving a typical maximum detection rate of 13.3 MHz [7]. This is small compared to the 60 MHz system clock speed of our digital correlator (Flex02-01D/C, Correlator.com), which was used in single channel photon history recording mode to obtain photon arrival timestamps. These photon arrival timestamps were then autocorrelated in software using the Laurence algorithm [37]. Our digital correlator was also used to autocorrelate photon arrival times in hardware using the multi-tau algorithm [20,38], using a fixed value of 106 delay times per decade by default. However, our implementation of the Laurence algorithm provides more flexibility as we can define in software both the delay times of the autocorrelation function and the frame rate of the DCS measurement.

**Fig. 5.**
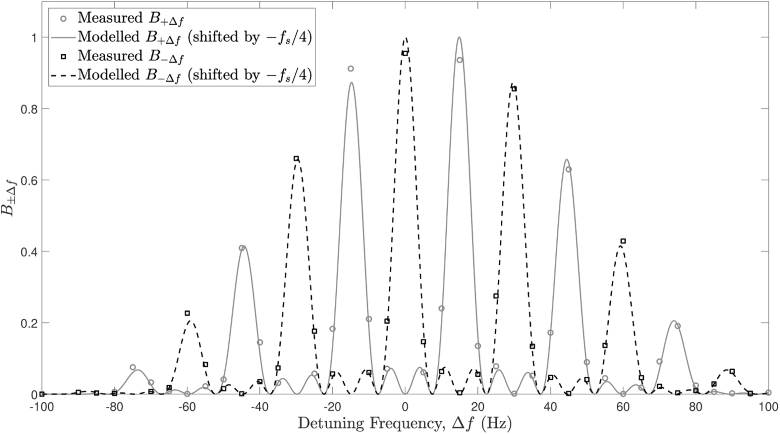
4-phase IRF detection and validation against the IRF model for the first and second heterodyne terms ($\tau_{e}=9.6$ ms, $f_{s}=29.8$ Hz, $N_{f}=4$).

In order to validate our holographic DCS instrument, as well as Eqs. (22) and (27), a series of 4-phase IRF measurements was collected using a static polyester resin optical phantom for -100 Hz ≤Δf≤ 100 Hz (Fig. 5). In this figure, each IRF model has been normalised by its respective maximum value. Each measured IRF data set has been normalised according to a least squares fit to the normalised IRF model (Eq. (22)), allowing for a constant noise offset in each case. The IRF models in Fig. 5 have been shifted by $-f_{s}/N_{f}$, as the peak sensitivity to the first heterodyne gain term of the IRF is at DC for phase-shifting holography. There is excellent agreement between measured and modelled data, which serves to validate our instrument’s design.

### 4.1 Mixed model fitting

This section describes the mixed motion model fitting process that was applied to measured DCS data in this study. Conventional DCS data ($g_{2}$) were fit to Eq. (1) by minimisation of the unweighted least squares objective function

(30) $$\text{arg min}\;\sum_{i=1}^{i=k}{\left[g_{2}{\left(\tau_{i}\right)}_{\text{measured}}-g_{2}{\left(\tau_{i}\right)}_{\text{model}}\right]}^{2}$$

over k delay times. This was performed separately for both Brownian and convective motion models (i.e., paired vectors of $[D_{b}$, $\beta_{D_{b}}]$ and $\left[\langle V^{2}\right\rangle$, $\beta_{\left\langle V^{2}\right\rangle }]$ were optimised for separately). In a third and final optimisation step, the contribution of each of these two models to a mixed motion model could then be determined by optimising for a ‘Brownian factor’ ($F_{Br}$), which is constrained to have a value between 0 and 1. This was achieved by minimisation of the unweighted least squares objective function

(31) $$\text{arg min}\;\sum_{i=1}^{i=k}{\left[g_{2}{\left(\tau_{i}\right)}_{\text{measured}}-\left[F_{Br}\times g_{2}{\left(\tau_{i}\right)}_{\text{Brownian}}+(1-F_{Br})\times g_{2}{\left(\tau_{i}\right)}_{\text{convective}}\right]\right]}^{2}.$$

**Fig. 6.**
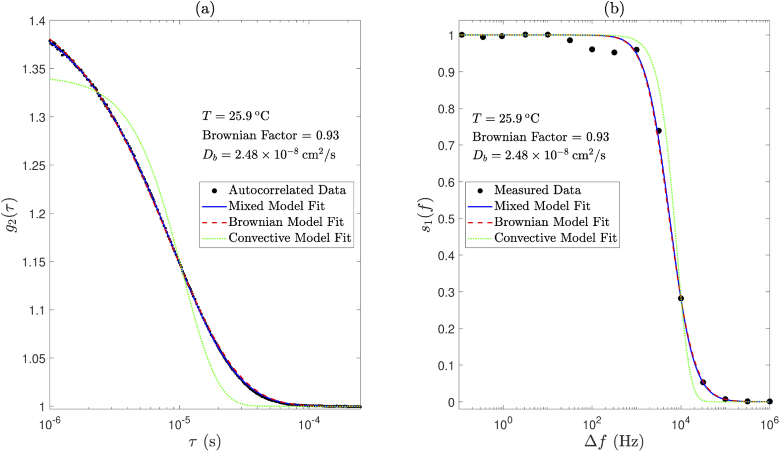
Application of (a) conventional DCS mixed model fitting and (b) holographic DCS mixed model fitting to data acquired using an intralipid phantom. Magnified views are shown in Fig. 7.

**Fig. 7.**
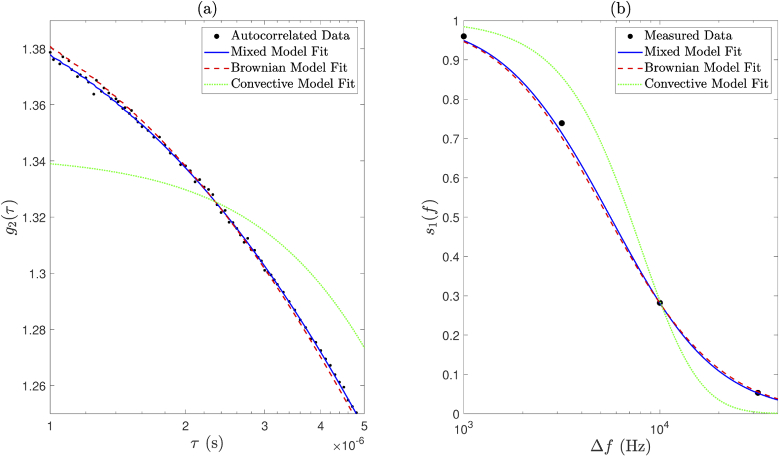
Magnified views of Fig. 6 show that the mixed model fits the data better than either the Brownian model or the convective model alone, for both (a) conventional DCS and (b) holographic DCS.

The Brownian model fit and the extracted $D_{b}$ value could then be scaled by the Brownian factor. Likewise, the convective model fit and the extracted $\left\langle V^{2}\right\rangle$ value could then be scaled by (1 - Brownian factor). The mixed motion model fit is then then sum of these two scaled model fits, and we assume that flow parameters scale linearly with changes in Brownian factor. An example of this mixed model fit is shown in Fig. 6(a) and Fig. 7(a).

Similarly, measured $S_{1}$ data were fit to Eq. (8); however, these data are implicitly un-normalised, and therefore fitting for a normalisation constant ($C_{norm}$) and noise floor parameter ($N_{floor}$) was also necessary. Flow parameters (i.e., $D_{b}$ or $\left\langle V^{2}\right\rangle$), $C_{norm}$ and $N_{floor}$ were optimised for by minimisation of the weighted least squares objective function

(32) $$\text{arg min}\;\sum_{i=1}^{i=k}w_{i}{\left[C_{norm}(S_{1d}{\left(\Delta \omega_{i}\right)}_{\text{measured}}-N_{floor})-s_{1d}{\left(\Delta \omega_{i}\right)}_{\text{model}}\right]}^{2},$$

over k detuning frequencies, where $w_{i}$ represents the weights. $s_{1d}{\left(\Delta \omega \right)}_{\text{model}}$ is the forward model normalised by its maximum value (i.e., its value at the smallest measured positive detuning frequency), and $w_{i}$ is calculated as $1/\sigma_{i}^{2}$, where $\sigma_{i}$ is the standard deviation of the measured data at the $i^{th}$ frequency step. Again, in a third and final optimisation step, the contribution of each of these two models of motion to a mixed motion model could then be determined by optimising for a Brownian factor (constrained to take a value between 0 and 1). This was achieved by minimisation of the weighted least squares function

(33) $$\begin{array}{rl} & \text{arg min}\;\sum_{i=1}^{i=k}w_{i}[C_{norm}(S_{1d}{\left(\Delta \omega_{i}\right)}_{\text{measured}}-N_{floor})-\cdots \\ & \;{\left[F_{Br}\times s_{1d}{\left(\Delta \omega_{i}\right)}_{\text{Brownian}}+(1-F_{Br})\times s_{1d}{\left(\Delta \omega_{i}\right)}_{\text{convective}}]\right]}^{2}. \end{array}$$

The Brownian factor could then be used to weight the contribution of each of the two model fits to the mixed motion model, and to scale the values of $D_{b}$ and $\left\langle V^{2}\right\rangle$ accordingly. An example of this mixed model fit is shown in Fig. 6(b) and Fig. 7(b).

### 4.2 Experiment 1 - absolute equivalence at room temperature

For the purposes of demonstrating absolute equivalence between conventional and holographic DCS techniques, we elected to use a sufficiently long camera exposure time (30 ms) together with a camera frame rate of 17.5 Hz, such that convolution of the true power spectra with the IRF could be ignored in the model fit process. This is the same camera exposure time that was used in [19] to accurately measure known convective flow rates *in vitro*, which were controlled using a calibrated syringe pump.

An intralipid optical tissue phantom (Intralipid 20 %, Fresenius Kabi) was prepared which consisted of 25.62 ml of intralipid made up to 550 ml with deionised water, resulting in optical properties of $\mu_{s}^{\prime }$ = 7.5 ${\mathrm{cm}}^{-1}$ (based on former in-house calibration measurements) and $\mu_{a}$ = 0.026 ${\mathrm{cm}}^{-1}$ at $20^{o}$C [39] (assuming that the optical absorption of intralipid is primarily due to background water absorption, $\mu_{a}^{BKG}$, as water is the main absorbing component of intralipid [40,41]). A temperature dependent model for the optical properties of combined intralipid/deionised water phantoms at 785 nm was constructed using the refractive index model of pure water presented in [42], the optical absorption coefficient model of pure water presented in [39], and the temperature coefficient for the reduced scattering coefficient of intralipid [40] (personal communication), having made scaling adjustments to allow for intralipid concentration differences.

The liquid phantom was contained within a glass beaker, which itself was immersed in a thermostatically controlled waterbath. It is important to control and account for the temperature of the phantom during absolute equivalence experiments, as not only does temperature affect the optical properties of the phantom, but it also affects the value of $D_{b}$ within the phantom, according to the Stokes-Einstein equation [19,28,43,44]

(34) $$D_{b}=\frac{k_{\text{B}}T}{6\pi \eta r},$$

where $k_{\text{B}}$ is Boltzmann’s constant, T is absolute temperature, η is dynamic viscosity, and r is the hydrodynamic radius of spherical particles diffusing through a liquid in the limit of a low Reynolds number. Furthermore, the dynamic viscosity of fluids also has a temperature dependence, and this was modelled according to the empirical fit

(35) $$ln\left(\frac{\eta }{\eta_{0}}\right)=a+b\left(\frac{T_{0}}{T}\right)+c{\left(\frac{T_{0}}{T}\right)}^{2},$$

where, for water, $T_{0}$ = 273.16 K, $\eta_{0}$ = 1.792 × $10^{-3}$ Kg/(m⋅s), and suggested coefficient values are a = -1.94 , b = -4.80 and c = 6.74 [45]. The SDS distance, as measured from the centre of the source fibre to the centre of the detector, was set to 17.5 mm.

**Fig. 8.**
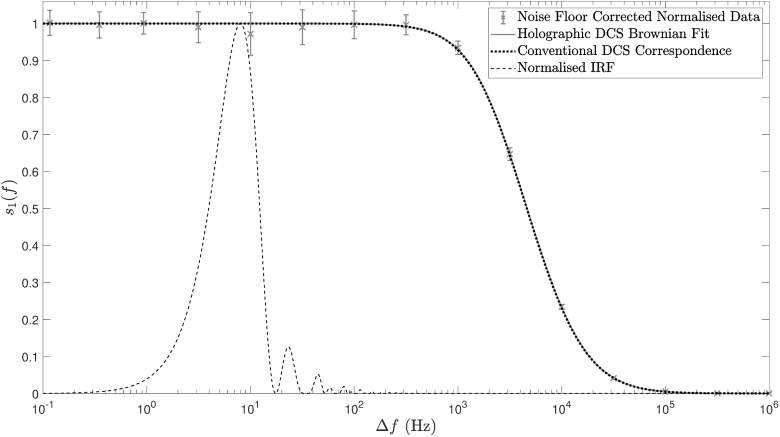
Fitting holographic DCS data to our Fourier domain DCS model (grey error bars and grey solid line), which is at least two orders of magnitude wider than the IRF (black dashed line). The black dotted line represents synthetic data produced by forward modelling in the Fourier domain with the $D_{b}$ value acquired from a conventional DCS setup.

**Fig. 9.**
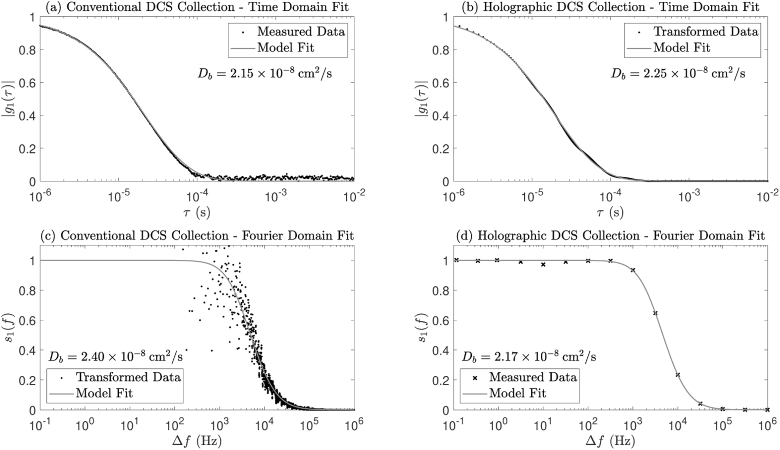
Fitting of measured data to native domain decorrelation models, for (a) conventional DCS collection and (d) holographic DCS collection. Model fitting in the complementary domain following numerical Fourier transform of measured data, for (b) holographic DCS collection and (c) conventional DCS collection. Due to noise in the measured data, DCS model fitting in the native domain is preferable to numerical transform and fitting in the complementary domain.

Holographic DCS data were gathered by implementing a logarithmic frequency sweep consisting of 15 steps between 0.1 Hz and 1 MHz, with 201 camera frames recorded at each frequency step, resulting in 200 data points at each detuning frequency for a DC subtraction temporal filtering method. The measured data were then fit directly to Eq. (8) using a Brownian motion model (as described by Eq. (32)) the results of which are depicted in Fig. 8 and Fig. 9(d). The former of these two figures shows ±1 standard deviation of the noise floor corrected and normalised data, together with the model fit to our Fourier domain DCS model of Brownian motion. The average level of assumed Gaussian noise (as a percentage of mean $s_{1}$ value) was calculated at each detuning frequency, the median of which was determined to be 3.8 %. The full width at half maximum (FWHM) of the measured signal is at least two orders of magnitude larger than the main lobe of the IRF FWHM, and we therefore neglect the effects of IRF broadening in this experiment. Our forward modelling simulations show that using these parameters, assuming 3.8 % Gaussian measurement noise averaged over 200 readings, an enforced condition of α=1 and a DC subtraction temporal filtering method, we expect a final $D_{b}$ estimation error of ∼0.14 %.

Conventional DCS data were gathered by collecting 30 seconds of multi-tau autocorrelated $g_{2}$ data, which were fit to Eq. (1) assuming α = 1 and a Brownian model of motion, the result of which is shown in Fig. 9(a). The extracted value of $D_{b}$ was input into our Fourier domain DCS forward model of Brownian motion, which is shown in Fig. 8 (black dotted line).

The naïve approach to recovering flow information from Fourier domain data is to numerically Fourier transform the data into the time domain, and to use established theory to fit for $D_{b}$. This approach is depicted in Fig. 9(b), and would at first glance be considered acceptable under the conditions of this experiment: a sufficiently narrow IRF, a sample composed entirely of dynamic scatterers, and the ability to average out noise over a generously large data set. However, due to the logarithmic spacing of the measured data, linear resampling prior to numerical Fourier transform is required (using shape-preserving piece-wise cubic interpolation with a resolution of 0.1 Hz in this case). This two-step process is not immune to the presence of noise in the measured data, which results in sub-optimal fitting in the complementary domain: the $D_{b}$ value acquired in Fig. 9(b) is not in keeping with those acquired by fitting in the native domains, which are shown in Fig. 9(a) and 9(d). This is confirmed by considering the time domain fit to the transformed Fourier domain native *model fit* (not shown), which produces a good $D_{b}$ value correspondence (2.15 × $10^{-8}$ ${\mathrm{cm}}^{2}$/s) to that of Fig. 9(a).

Similarly, conventional DCS data were transformed into the Fourier domain, following linear resampling with a resolution of 0.5 μs, and were fit to our Fourier domain DCS model of Brownian motion, the result of which is shown in Fig. 9(c). The value of $D_{b}$ produced by this model fit is also not in keeping with that acquired by fitting in the native domains; however, this can be rectified by considering the Fourier domain fit to the transformed time domain native *model fit* (not shown), which yields a much closer $D_{b}$ value correspondence (2.13 × $10^{-8}$ ${\mathrm{cm}}^{2}$/s) to that of Fig. 9(d).

### 4.3 Experiment 2 - absolute equivalence over temperature

To show the absolute equivalence of conventional and holographic DCS techniques over a physiologically relevant temperature range, as well as to demonstrate ground-truth validation from other measurement techniques, we repeated Experiment 1 at 12 temperature steps during heating between $17.1^{o}$C and $40.5^{o}$C. According to Eq. (34), and using our temperature dependent model of the optical properties and dynamic viscosity of intralipid, the $D_{b}$ value of the optical phantom will increase in a non-linear fashion as its temperature is increased. Intralipid optical phantoms have previously been demonstrated to have good thermal stability, with scattering properties varying less than 0.5 % when held at $70^{o}$C for 12 hours, and are therefore considered to be optically robust and stable when subject to elevated temperatures [46].

A thermostatically controlled waterbath was allowed to stabilise for 30 minutes before collecting each data set [40], and the phantom was manually stirred after each temperature increase in order to maintain homogeneity. Evaporative losses are to be expected when performing such an experiment, as such the optical probe was lowered slightly as need be before each measurement, in order to ensure good optical coupling with the phantom. Loss of water from the phantom will also alter its optical properties, by way of increasing the concentration of scatterers and decreasing the water concentration [41]; however, we deemed this effect to be minimal in our experimental setup due to both the original volume of the phantom and the relatively short span of time over which data were collected following the lowering of the optical probe.

**Fig. 10.**
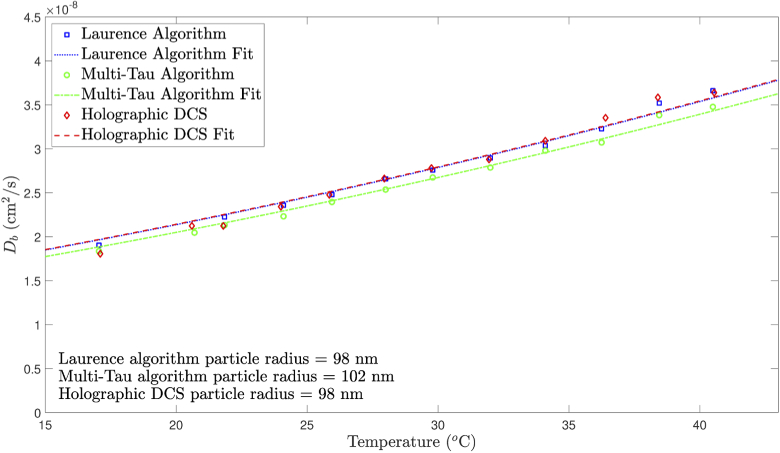
The distribution of $D_{b}$ values for both conventional DCS and holographic DCS over a temperature range in an optical tissue phantom using native domain mixed model fitting. Model fits to the Stokes-Einstein equation and extracted intralipid particle radii are also shown for all three data sets.

In addition to the data acquired in Experiment 1, 15 seconds of raw photon counting data were also collected at each temperature step. These data were autocorrelated using our implementation of the Laurence algorithm. Autocorrelated $g_{2}$ data were then fit to our conventional DCS mixed model, with the distribution of the mixed model fit $D_{b}$ values being displayed in Fig. 10 (this figure also shows the corresponding multi-tau autocorrelated $D_{b}$ data). The 200 holographic DCS frequency sweeps were averaged, and the $D_{b}$ values acquired by fitting to our Fourier domain mixed motion model are shown in Fig. 10.

Figure 10 also shows the results of fitting the mixed motion model $D_{b}$ values to the Stokes-Einstein equation, both in terms of the Stokes-Einstein model fits and the extracted intralipid particle radii, for all three data sets. There is close agreement between the Laurence algorithm, multi-tau algorithm and holographic DCS data, which yield an intralipid particle radius of 98, 102 and 98 nm, respectively. These values are all within a maximum 4 % deviation of each other. Furthermore, these values are in close agreement to previous measurements of intralipid by the same manufacturer using transmission electron microscopy (TEM) in [47], which suggest an average particle radius of 107 nm. By failing to account for a mixed model fit in this experiment (i.e., by using a Brownian motion model only, not shown), intralipid particle radii of 90, 94 and 88 nm are extracted for the Laurence algorithm data, multi-tau algorithm data and holographic DCS data, respectively.

### 4.4 Experiment 3 - SNR scaling and insensitivity to ambient light

**Fig. 11.**
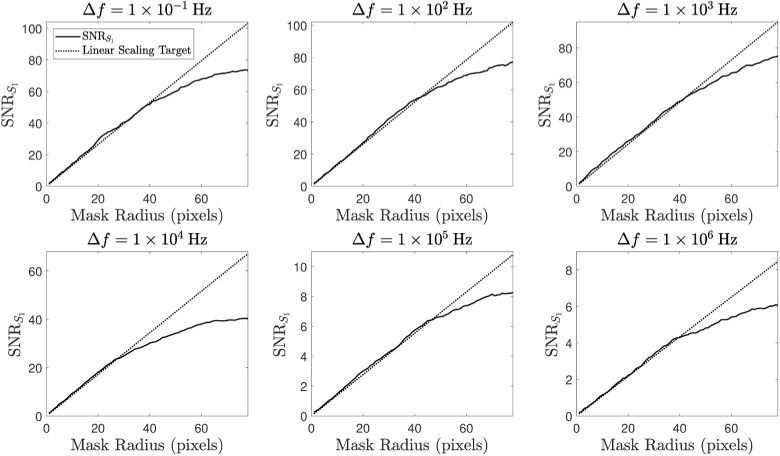
The relationship between ${\text{SNR}}_{S_{1}}$ and the radius of the demodulation mask at various Δf values for holographic DCS, the linear scaling targets are shown by the dotted lines.

The SNR of a speckle detection system should scale linearly with the square root of the number of speckles detected [48]. We therefore sought to verify this for the raw $S_{1}$ values produced by our instrument, using the same optical phantom as Experiment 1. Camera parameters of $f_{s}$ = 100 Hz and $\tau_{e}$ = 0.3 ms were chosen for this experiment. Here we define the SNR in $S_{1}$ to be

(36) $${\text{SNR}}_{S_{1}}=\frac{\mu (S_{1})}{\sigma (S_{1})},$$

with a sample size of N=500 values in this case. By varying the size of the reconstruction mask in the holographic demodulation process, we can effectively control the number of speckles that contribute to each $S_{1}$ measurement. The resulting SNR values are plotted in Fig. 11, where we expect to observe a linear fit between SNR and mask radius in all 6 subplots, which correspond to different Δf values (the square root number of modes is a linear function of mask radius). Instead, the SNR of $S_{1}$ appears to form an asymptote, which suggests another source of noise becomes limiting. We believe that this effect is in part due to temporal noise in the intensity of the laser source, $S_{0}$, which introduces fluctuations into the measured $S_{1}$ data (according to Eq. (8)).

We also define the SNR of a $D_{b}$ measurement to be the mean $D_{b}$ value over N measurements, divided by the standard deviation in those measurements

(37) $${\text{SNR}}_{D_{b}}=\frac{\mu (D_{b})}{\sigma (D_{b})}.$$

To assess the ${\text{SNR}}_{D_{b}}$ benefit conferred by our instrument under optical blackout conditions, we measured ${\text{SNR}}_{D_{b}}$ (using a Brownian model of motion) over a range of flow parameter output rates in an intralipid phantom with optical properties similar to that of brain tissue ($\mu_{a}$ = 0.10 ${\mathrm{cm}}^{-1}$ and $\mu_{s}^{\prime }$ = 7.5 ${\mathrm{cm}}^{-1}$ [7]), for an SDS distance of 15 mm and N=100. This was achieved by the addition of Indian ink (Windsor & Newton, Liquid Indian Ink, 1010754) to an intralipid phantom, based on in-house dilution calibration experiments (using a PerkinElmer Lambda 750 S UV/Vis/NIR Spectrophotometer). By varying the number of camera frames taken at each detuning frequency, we can effectively trade the overall parameter output rate for the number frames to average, and thus ${\text{SNR}}_{D_{b}}$ in the measurement. For example, with measurement at 6 detuning frequencies, a camera exposure time of 0.3 ms and a camera frame rate of 303 Hz, we can obtain an overall parameter output rate of 23.8 Hz by capturing 2 camera frames per detuning frequency. Increasing the number of frames captured per detuning frequency to 11 effectively decreases the overall parameter output rate to 4.5 Hz.

We then performed equivalent analysis on conventional DCS data (autocorrelated using the Laurence algorithm) collected under matched conditions by collecting raw photon counting data and discretising it into N samples (each of length equal to $T_{frame}$, the *total* time required to acquire a $D_{b}$ frame using our holographic DCS instrument). Using a Brownian motion model fit to this data, we then calculated an ${\text{SNR}}_{D_{b}}$ value for conventional DCS data at matched overall parameter output rates. For both holographic DCS and conventional DCS data, we observed that ${\text{SNR}}_{D_{b}}$ scales with $\sqrt{T_{frame}}$ (as is to be expected for a variable exhibiting Gaussian noise).

Our holographic DCS instrument offered an improvement in ${\text{SNR}}_{D_{b}}$ over conventional DCS by a mean factor of 2.3 over all 10 parameter output rates that were investigated. However, repeating this experiment under normal ambient lighting conditions increased this ${\text{SNR}}_{D_{b}}$ mean improvement factor to 5.3, due to degradation of ${\text{SNR}}_{D_{b}}$ associated with the conventional DCS technique. This confirms the relative insensitivity of the holographic DCS technique to ambient light. We note that the camera operated with a duty cycle of only 9.1 % during this ${\text{SNR}}_{D_{b}}$ comparison, and we are therefore motivated to increase this duty cycle in future work in order to further increase the SNR advantage of our system (this could be achieved using a multiple camera setup, for example).

### 4.5 Experiment 4 - *in vivo* feasibility

The feasibility of making *in vivo* measurements with our instrument was demonstrated by acquiring forearm contact measurements. This study was approved by the UCL Research Ethics Committee, project ID number: 1133/001. Our *in vivo* probe used an SDS distance of 11.3 mm and the beam was expanded in order to adhere to patient safety limits [49]. By developing an understanding of the complex interplay between the parameters of our instrument and the flow values we expect to encounter in a particular DCS geometry, we are able to determine the most appropriate camera exposure parameters for any given experiment. For example, when making forearm measurements we assume optical properties of $\mu_{a}$ = 0.25 ${\mathrm{cm}}^{-1}$ and $\mu_{s}^{\prime }$ = 4.27 ${\mathrm{cm}}^{-1}$. These are the average of the measurements acquired from the forearm of three healthy volunteers in [7] at a wavelength of 788 nm. This group used sample optical properties measured at 788 nm to analyse data from DCS experiments of the same samples, which were undertaken using a 785 nm laser source. We therefore deem the values that we have selected to be appropriate estimates of the optical properties of our sample in this experiment, which also operates at 785 nm.

**Fig. 12.**
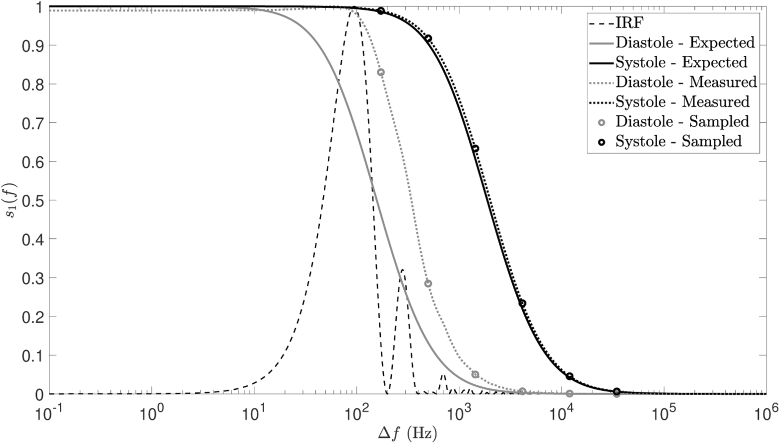
Simulated data, used in Experiment 4 to aid selection of appropriate detuning frequencies. The solid lines show the range of power spectra that we expect to encounter (using $D_{b}$ values acquired from conventional DCS data collection). The dotted lines simulate the effects of IRF broadening on the expected power spectra. The measured power spectra are then sampled where we expect to encounter the greatest measured change over the cardiac cycle (these sample points are depicted by the circles on each of the measured power spectra).

Through the use of a conventional DCS system, these optical properties were used to recover the range of $D_{b}$ values that we would expect to measure from our sample. This allowed us to model the power spectra that we would expect during both diastole and systole for Brownian motion, as shown by the grey and black solid lines in Fig. 12, respectively.

**Fig. 13.**
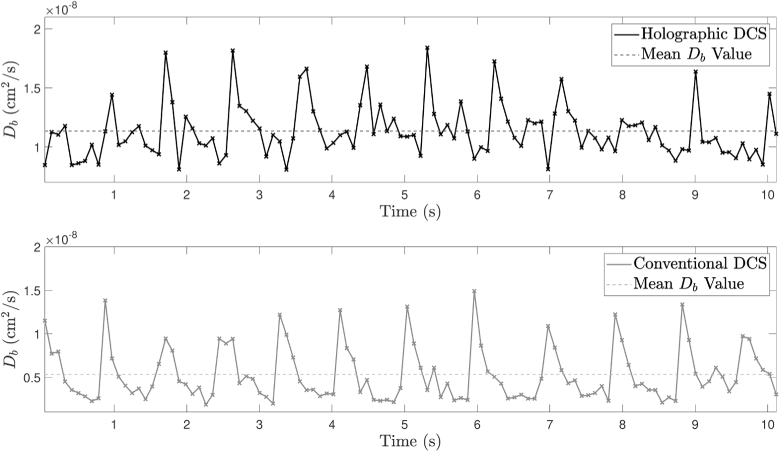
$D_{b}$ time series for contact forearm measurements acquired at 10.8 Hz, using both holographic DCS and conventional DCS. The dashed horizontal lines represent the mean $D_{b}$ value for each time series.

Inspection of Fig. 12 shows that, for a camera exposure time of 2 ms and a camera frame rate of 200 Hz, the FWHM of the expected diastolic power spectrum and the FWHM of the IRF are equivalent, resulting in significant broadening of the measured diastolic spectrum (grey dotted line); however, this effect is much less pronounced during systole (black dotted line). We have chosen to sample at frequencies where the greatest measured change is expected over the cardiac cycle, and these are indicated by the circles on each of the measured power spectra in Fig. 12. Additionally, since the camera exposure time is in the order of the expected tissue decorrelation time (∼1 ms [9]), we expect optimal detection sensitivity [30]. Choosing the 6 frequency points shown, with three camera frames at each point, gives an overall parameter output rate of 10.8 Hz for these camera exposure parameters, ensuring that we can accurately recover pulsatile information (which we expect to contain significant frequency content at 1-2 Hz). This is validated by Fig. 13, which also shows equivalent conventional DCS data (autocorrelated using the Laurence algorithm). Fourier transforms of these $D_{b}$ time series, which were acquired using a Brownian model of motion, reveal peak content at 65 beats per minute in both cases. This was consistent with the resting heart rate of the volunteer in this study.

We note that the mean value of the holographic DCS $D_{b}$ time series in Fig. 13 is 1.13 × $10^{-8}$ ${\mathrm{cm}}^{2}$/s, whereas the mean value of the conventional DCS $D_{b}$ time series in the same figure is 0.58 × $10^{-8}$ ${\mathrm{cm}}^{2}$/s. We attribute this to broadening of the measured signal by the IRF of our system (Fig. 12), which artificially elevates the lower $D_{b}$ values especially (as confirmed by fitting $D_{b}$ values to the simulated measured data in Fig. 12).

## 5. Discussion and conclusion

The experiments performed in this paper have shown that our holographic DCS system can measure data that are entirely equivalent to a conventional DCS system, but with a higher optical throughput, a decreased cost of detector and a robustness to the effects of ambient light. Improved SNR has been demonstrated *in vitro*; however, this advantage is yet to be demonstrated *in vivo*. By developing and validating a mixed motion DCS frequency domain model, we are able to achieve accurate interpretation of the data produced by our instrument in its native domain, which is fundamentally different to conventional DCS data. These differences arise due to the alternative sampling strategies of data in the time and frequency domains, the effects of static scattering in a holographic DCS system, the nature of the noise, and broadening by the IRF, especially at high parameter output rates. These high output rates have been made possible by the design of our custom instrument with a high throughput and minimal dead-time, which enables highly parallel GPU-accelerated holographic demodulation and is thus suited to *in vivo* application.

With reference to Fig. 9, we conclude that, due to the noise in the measured data, DCS model fitting in the native domain is preferable to numerical transform and fitting in the complementary domain. Whilst the deviations in $D_{b}$ measurement using complementary domain fitting that are shown in Fig. 9 are relatively small for the very slow acquisition times used in the exemplar configuration of Experiment 1, they are significant when we optimise for fast acquisition times with a broader IRF, especially when imaging samples with a static scattering component. Thus fitting with a native model appears to be an appropriate technique to achieve accuracy in our flow parameter measurements.

Our mixed model fitting procedure has been rigorously validated by the results of Experiment 2. Compared to the Brownian model of motion, the mixed motion model $D_{b}$ values shown in Fig. 10 have an improved fit to the Stokes-Einstein relationship (Eq. (34)), and the extracted intralipid particle radii values are also in closer agreement with the TEM gold standard measurement [47]. This is because the sample becomes less Brownian and more convective as the temperature of the waterbath increases, and therefore allowing for this variation is important in revealing the underlying *absolute* $D_{b}$ agreement between the conventional and holographic DCS techniques. We therefore conclude that in order to show absolute equivalence between conventional DCS and holographic DCS over a temperature range, the use of a mixed model fit to account for a Brownian factor variation is essential. However, Experiments 1, 3 and 4 do not make use of mixed model fitting, as we do not expect the Brownian factor of the sample to vary within the course of each of these experiments.

Holographic DCS is an inherently heterodyne and multi-speckle detection technique. Conventional DCS has, until recently, been a homodyne single speckle detection technique. However, due to recent developments in the field [13,14,50], we note that a fuller comparison of our method (especially with regard to assessing SNR performance and robustness to ambient light) could be achieved by comparing holographic DCS to both single and multi-speckle conventional heterodyne DCS techniques (i.e., iDCS), and these comparisons will be addressed in our future work. However, we note that the reduction in detector cost afforded by holographic DCS, as well as the scalability of a detection strategy that operates at the shot noise limit, is a compelling advantage of our technique.

Despite validating and demonstrating the potential advantages of holographic DCS, this paper does not reflect the full potential of this technique. The short camera exposure times that were used in Experiment 4, and which are necessary to sample fast enough so as to resolve pulsatile information, introduce a complication to our measured data. Spectral broadening by a relatively wide IRF artificially elevates the measured $D_{b}$ values in Fig. 13. Correcting for this, whilst sampling at only a limited number of detuning frequencies (especially in the presence of static scattering due to the skull, for example), represents a significant challenge and this will form part of our future work. Following on from this, the SNR advantage that was demonstrated *in vitro* in Experiment 3 was not achieved *in vivo* in Experiment 4. We suspect that this is in part due movement artefact (which is a known problem of multimode detection [50]) and sub-optimal tissue coupling in our system [6]. Therefore experimentation into the effect of various collection optics and tissue coupling mechanisms, together with minimising other sources of movement and vibration in our system, is warranted.

Whilst we note that the SDS distances used in this study (1.13 - 1.75 cm) are relatively short compared to the SDS distances of 2 - 3 cm typically used in human applications [1], this does not detract from the purpose of this proof of concept study, where our aim is to demonstrate the quantitative equivalence of measurements made using the holographic DCS method. Our future work will assess the feasibility of our system to detect signals under the more challenging conditions provided by larger SDS distances.

Our future work will also involve overcoming the issues encountered with SNR scaling and duty cycle optimisation that were highlighted by Experiment 3. Validation of an SNR advantage is a necessary precursor to enhancing the spatial resolution and imaging depth of our instrument using ultrasound modulation. Our holographic DCS system is currently limited by the duty cycles available with our camera, especially when using very short camera exposure parameters. Figure 11 shows that the ${\text{SNR}}_{S_{1}}$ values at all detuning frequencies scale linearly with the radius of the demodulation mask up to a certain point, before another source of noise is encountered that becomes limiting. We have confirmed that the measured speckle patterns do indeed conform to the negative-exponential probability distribution function expected of a fully developed speckle pattern [35], and preliminary modelling has shown that realistic values of RMS intensity noise on the laser source could account for the asymptotic nature of the ${\text{SNR}}_{S_{1}}$ curves in Fig. 11. Indeed, it has previously been observed that laser instability has a larger influence on DCS measurements when using heterodyne techniques [50]. Assessment of the laser temporal intensity spectrum (with stabilisation of the laser amplitude output as necessary) as well as more sophisticated techniques to remove sources of temporal noise from measured holograms (e.g., an eigenvalue decomposition and filtering approach to remove parasitic noise sources [51]), could be investigated in order to solve this problem.

To the authors’ knowledge, this paper represents the first time that a holographic DCS technique has been used to recover *in vivo* flow measurements, at a fast enough sample rate to ensure the accurate recovery of pulsatile information. Additionally, this technology can readily be applied to longer wavelengths, which have previously been shown to improve SNR and depth sensitivity, but which are currently incompatible with existing detector technologies in conventional DCS. This offers exciting prospects not only for the potential of deeper DCS measurements, but also for the potential of acquiring spatially resolved DCS measurements using AOT hybrid techniques.
